# Assessing the impact of the eco-environmental damage compensation system

**DOI:** 10.1038/s41598-025-12241-x

**Published:** 2025-08-19

**Authors:** Xiaoqi Wu, Yawei Feng

**Affiliations:** 1 School of Economics and Management, Northwest University, Xi’an, Shaanxi China; 2 XAUAT Engineering Technology Co., Ltd, Xi’an, Shaanxi China

**Keywords:** Eco-environment damage compensation system, Ecological and environmental level, Resource-based cities, Industrial structure, Technological innovation, Climate-change policy, Climate-change mitigation, Environmental sciences, Environmental social sciences

## Abstract

The high-quality development path of ecological priority and green energy efficiency should be unswervingly followed. Therefore, exploring the ecological and environmental level (EEL) is an urgent and essential matter for sustainable development. This research aims to estimate how the eco-environmental damage compensation system (EDCS) affects the EEL in China. The framework is based on panel data collected from 284 cities at the prefecture level in China. The Differences-in-Differences (DID) method is employed to examine the influence and spillover effect. Findings provide evidence that the EDCS significantly improves the EEL. Therefore, this study primarily examines whether and how the EDCS impacts the EEL. Further, the ecological environment level of resource-based cities is drastically different from that of non-resource-based cities under the impact of the EDCS. Mechanistic analyses demonstrate that the EDCS is conducive to upgrading of industrial structure and technological innovation, which in turn promotes regional EEL. Based on the findings of the analyses, it can be argued that policymakers and market participants should work together to address environmental issues, combining top-down and bottom-up approaches. Ultimately, efficient markets and responsive governments work together. The policy should form a dynamic adjustment mechanism for different resource cities to realize the purpose of emissions reduction and enhance the EEL.

## Introduction

China’s economy has experienced sustained high growth since the reform and opening up of the country. China has emerged as the global leader in energy consumption and carbon emissions due to its remarkable economic development pace^1^. The acceleration of industrialization and urbanization has been accompanied by a deteriorating environment and declining air quality, which have threatened people’s health. At present, China’s economic development has entered a new normal, which has shifted from high-speed growth to high-quality development, and it has become a topic to promote the optimization of China’s ecology and environment and to construct an ecological civilization institutional system^2^. The EDCS is an integral part of the ecological civilization system. In order to solve the problem of not being compensated for severe environmental pollution and ecological damage losses caused by polluters and not protecting the public’s environmental rights, interests, and ecosystems, the General Office of the Communist Party of China Central Committee and the General Office of the State Council jointly issued the reform plan for the EDCS in 2015, and deployed pilot program in seven provinces and cities including Jilin, Jiangsu, Shandong, Hunan, Chongqing, Guizhou, and Yunnan. They have been implementing the EDCS nationwide since 2018.

‘Enterprises pollute, the public suffers, and the government pays the bill’ has always been a pain point. The cost of compliance for Chinese enterprises is too high, while the cost of violating a law is too low. The existing legal system and framework are ineffective in solving this problem. The EDCS has made up for a gap in the current law and system by giving the power and responsibility to act as the owner of the ecology and environment under its jurisdiction and allowing negotiation or lawsuits when ecology and environment damage occurs. The stability of China’s political system facilitates the effective implementation of the EDCS^3^. According to statistics, since the implementation of the EDCS in the past 2 years, 27 cases of ecology and environment damage compensation have been initiated, involving an amount of about 401 million yuan, of which 13 have been negotiated for the compensation of ecology and environment damage. Nine have reached an agreement on the negotiation, while the remaining 14 cases are in the process of judicial appraisal, which causes a social sensation. However, this study is not concerned with the impact of the EDCS on the EEL at the stage of judicial litigation. On the contrary, our article focuses on the synergistic effect, which examines the negative impact of PM2.5.

Research on the EDCS can better explore the path to realizing the ‘dual-carbon’ goal and help China make the ‘China Commitment’ at the United Nations Climate Change Conference, which has a profound impact on global environmental governance. This commitment to reduce emissions adheres to the new development concept, will vigorously promote the world’s sustainable development, and has attracted widespread attention from scholars all over the world. As the environment continues to deteriorate, seriously affecting people’s livelihoods, the government is forced to carry out pollution prevention and control, and ultimately, the formation of a ‘pollution first, then governance’ passive situation. There are many ways to solve the negative environmental externalities. According to Coase’s Theorem, clear property rights are needed to realize the efficient distribution of resources. Although clear initial property rights allocation is a prerequisite for solving positive externalities based on market negotiation^4^, it is difficult to define property rights, and the ‘tragedy of the commons’ occasionally occurs. Although the use of regulation can promote technological progress, control environmental pollution, and achieve economic growth^5–12^, from the perspective of the whole society, it hinders market operation, thus causing a loss of efficiency^13^. The emissions trading system in market economy countries in the field of environmental protection is a specific application of the Coase’s Theorem^14^, and the mechanism of emissions trading rights can effectively curb pollutant emissions, which can significantly promote emission reduction^15–18^, the emissions trading system can promote regional industrial structure upgrading^19,20^ and outward foreign direct investment^21,22^, but if the market mechanism is imperfect and the market transaction cost is high, it will also ultimately result in the failure of Coase’s theorem and put an excessive burden on the enterprises^14^, and Zhang and Xiang^23^ found that SO_2_ emissions trading significantly increased the degree of environmental inequality in the pilot region. The EDCS can reverse this passive situation. Like the Pegu tax, the government can achieve optimal externality by implementing the EDCS^24^. The EDCS internalizes the externality of ecological damage and environmental pollution and achieves the paid use of resources through user fees to protect the environment^25^ and mitigates environmental pollution and thus reach environmental sustainability^26^.

Domestic scholars on the EDCS focus more on the concept, theory^27,28^, and legal research, specifically including research on the legal system^29^, research on consultation in the compensation stage^30^ and research on later litigation^31–33^. However, there is less literature related to the evaluation of the EDCS. Gao^34^ measures the dynamic effect of the EDCS under different shocks through DSGE. This study focuses on the EDCS and constructs the EEL indicator to measure its power. This study will provide a deeper understanding of the influence of the EDCS and give a reference for realizing China’s ‘dual-carbon’ target path.

This study aims to explore the policy impact on the EEL since the pilot of the EDCS and to test whether the EEL of cities has been improved in the pilot provinces at this stage. This article uses the entropy method to construct the EEL indicator for measuring the comprehensive score of the ecology and environment. Therefore, this study primarily examines whether and how the EDCS impacts the EEL, endeavoring to address the following questions: Firstly, can this system facilitate the enhancement of the EEL? Secondly, what is the mechanism through which the EDCS influences the EEL?

**Fig. 1:**
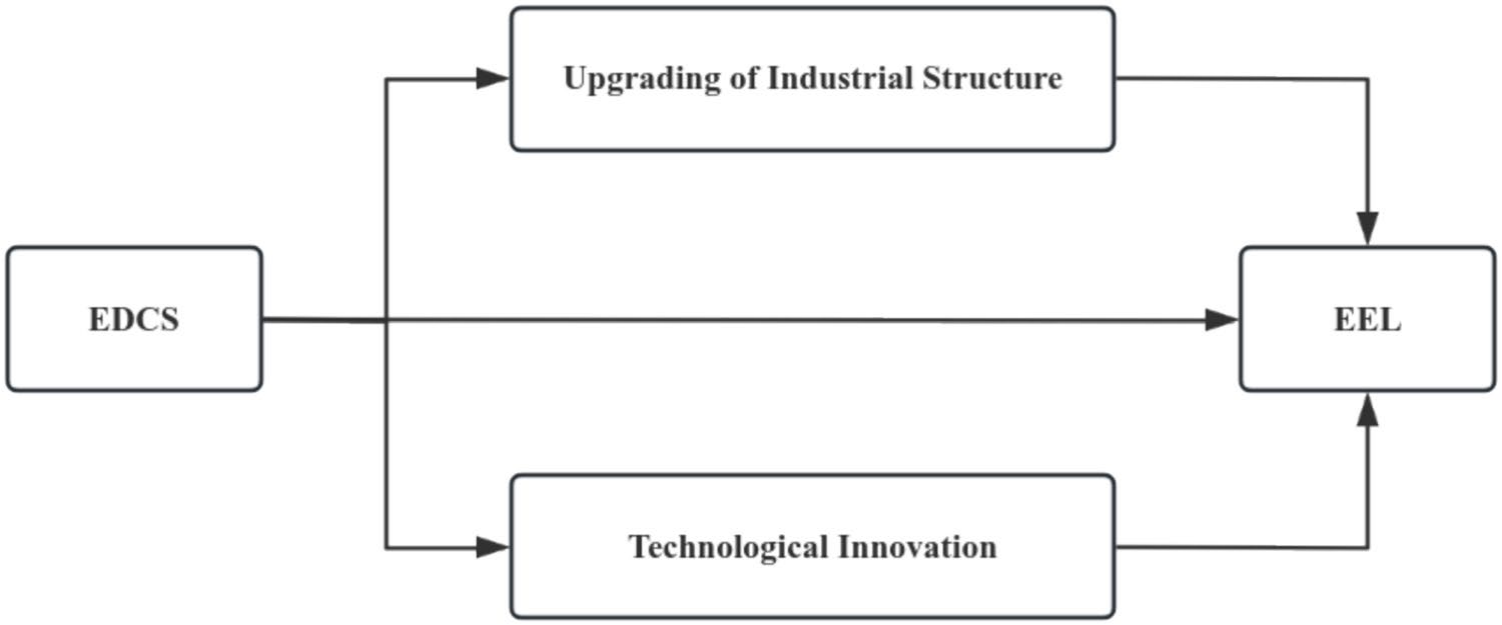
The impact mechanism of the EDCS.

This article initially delves into the policy implications of the EDCS on emission reduction since its pilot implementation, examining whether this policy has effectively enhanced the EEL of cities in the pilot provinces by focusing on these urban areas as our research subjects. Subsequently, we explore the underlying mechanisms at play. Drawing upon Porter’s innovation compensation hypothesis, we posit that a well-calibrated EDCS can spur corporate innovation, thereby boosting production efficiency, cutting costs, and enhancing the role of technological innovation in environmental governance^1^. Consequently, this hypothesis contends that appropriate environmental regulations not only elevate corporate production efficiency and market competitiveness but also mitigate environmental pollution. Furthermore, the EDCS facilitates the internalization of externalities, compelling enterprises to undergo structural upgrades. To better adapt to and meet shifting market demands, enterprises must transform their production methods and adjust their product mix, exemplifying the industrial structure transformation effect triggered by demand-side influences in Fig. 1.

Specifically, based on China’s urban environmental quality data from 2009 to 2017, we construct a DID model to study the impact of the EDCS on urban EEL. Further, the grouped regression method is utilized to examine the heterogeneity of the shocks of the EDCS on the EEL. The findings of this article show that the EDCS improves the EEL in the cities of the pilot provinces. The EDCS affects the EEL through two channels: upgrading of industrial structure and technological innovation. Compared with non-resource cities, the EDCS considerably increases theEEL in resource-based cities. Ultimately, it has contributed to realizing China’s ‘dual-carbon’ goal.

Compared with the existing studies, this article may be innovative in that it utilizes causal identification to study the power of the EDCS at the city level. While existing studies focus more on the rationality of the system design and the rule of law theoretical system supported by jurisprudence, the study quantitatively researches the impact of the EDCS on the environment and economic development from the mathematical model and empirical analysis. Secondly, it further measures the degree of influence of the EDCS on the EEL from the perspective of governmental behavior. It also helps to clarify the role of the relevant state departments under the EDCS and provides practical experience on how the ‘active government’ should act under the goal of ‘dual-carbon.’ Thirdly, this study constructs environmental level indicators based on the entropy value method to measure the influence of the EDCS, which enriches the literature related to environmental policy assessment.

The remainder of this article is organized as follows. The mathematical model and research hypothesis are described in “Literature review” section. “A quantitative model” section is the research design, which sequentially introduces sample selection, data sources, variables, data description, and model specification. “Research design” section is the empirical analysis and robustness test, which is based on the estimation results of the DID model for the practical examination of the EEL of the EDCS. The article concludes with “Empirical results and robustness tests” section.

## Literature review

The early literature on the EDCS primarily focused on conceptual definitions, theoretical foundations, and institutional frameworks. For instance, Liu^27^ and Yu et al.^28^ concentrated on these foundational aspects but neglected policy evaluation. Subsequent studies by Yu et al.^25^ and Zhang et al.^29^ explored legal dimensions but did not address economic assessments. Some scholars examined negotiation mechanisms during the compensation phase^30,31^ or litigation-related aspects post-compensation^31–33^, but these works did not investigate the deterrence effect of the policy or its impact on ecological quality. Gao^34^ used a DSGE model to simulate the dynamic effects of the EDCS under different shocks but limited the analysis to a macro-level perspective, omitting micro-level empirical evaluations.

Regarding ecological quality measurement, scholars have proposed numerous methods, but few have addressed dimensions such as objectivity and computational simplicity. Li et al.^8^ used a DEA method to calculate regional ecological efficiency in China from 1997 to 2010, but DEA results are susceptible to indicator selection. Jing et al.^35^ employed a three-stage DEA model for spatiotemporal comparisons of ecological efficiency across Chinese provinces from 2008 to 2017, incorporating environmental variables such as population density and GDP. However, this approach remains susceptible to subjective influences. Cheng^36^ developed an ecological civilization evaluation index system using principal component analysis (PCA) to synthesize indicators, but PCA, as a dimensionality-reduction technique, retains only the majority of the variance. Zhou et al.^37^ applied a weighted TOPSIS method to evaluate ecological quality in the Yangtze River Economic Belt but relied on subjective weighting, limiting objectivity. Wang and Li^10^ established an ecological quality index system for impoverished regions using the analytic hierarchy process (AHP), but AHP is prone to subjectivity and cognitive biases. Zuo et al.^38^ developed an ecological security evaluation framework based on the PSR model but faced challenges in indicator selection, weight determination, and data acquisition.

This study addresses these gaps by focusing on the EDCS and its policy effects on ecological quality from a micro-level perspective. Using an entropy method, we construct an ecological quality index based on four indicators across two dimensions: ecological resources and environmental quality. Additionally, we employ a difference-in-differences approach to evaluate the spillover effects of the EDCS in pilot regions, emphasizing its deterrence role and policy impact rather than effects during the judicial litigation phase. This work enriches the quantitative analysis of policy effects related to the EDCS.

## A quantitative model

### Constructing the ecological and environmental level

Drawing on Ren et al.^39^, the definition of the indicator of the ecological environment includes maintaining the integrity of ecosystem function, continuously improving the ecological environment to establish a new way of coordinating the relationship, maintaining the existing ecological environment, protecting the ecological diversity, and continuously improving the quality of the environment. Therefore, this study sets the EEL as two significant indicators of ecological resources and environmental quality indicators, including four secondary indicators, such as greening coverage of urban built-up areas, industrial sulfur dioxide emissions, industrial nitrogen oxide emissions, industrial smoke, and dust emissions. Through the original data matrix normalization, defining entropy, we calculate the composite index of the EEL.

The specific steps are as follows:

1. Derive positive indicator $positiveR_{ij}$ and negative indicator $negativeR_{ij}$ through matrix normalization.
  1 $$positiveR_{ij} = \frac{{A_{ij} - \mathop {\min }\limits_{j} \left\{ {A_{ij} } \right\}}}{{\mathop {\max }\limits_{j} \left\{ {A_{ij} } \right\} - \mathop {\min }\limits_{j} \left\{ {A_{ij} } \right\}}},$$
  2 $$negativeR_{ij} = \frac{{\mathop {\max }\limits_{j} \left\{ {A_{ij} } \right\} - A_{ij} }}{{\mathop {\max }\limits_{j} \left\{ {A_{ij} } \right\} - \mathop {\min }\limits_{j} \left\{ {A_{ij} } \right\}}},$$
  where $A_{ij}$ is the *j*^*th*^evaluation object of the *i*^*th*^ evaluation index. There are m evaluation indicators and n evaluation objects to form a data matrix.
2. Define the entropy of the *i*^*th*^ indicator $s_{i}$.
  3 $$s_{i} = - y\sum\limits_{j = 1}^{n} {f_{ij} \ln f_{ij} } ,$$
  where, $y = 1/\ln n$, *f*_*ij*_=*R*_*ij*_∕Ʃ*R*_*ij*_.
3. Define the entropy weight $w_{i}$.
  4 $$w_{i} = \frac{{1 - s_{i} }}{{m - \sum\nolimits_{i = 1}^{m} {s_{i} } }}.$$
4. Calculate the ith ecosystem composite indicator.
  5 $$C_{i} = \sum\nolimits_{j = 1}^{n} {w_{i} R_{ij} } .$$

The EDCS can internalize the externality enough to reduce the degree of ecological environment pollution, deter the behavior of ecological environment damage, reverse the degradation of the ecological environment, continue to improve the quality of the ecological environment, enhance the EEL, and play a particular role in maintaining the integrity of the ecosystem.

### An eco-damage compensation system model

Drawing on Forster^40^, we assume that, in a perfectly competitive market, individuals increase positive effects through energy consumption (C(E)) and adverse impact through environmental pollution (P) in order to maximize their utility (U). The marginal utility of consumption is positive and decreasing, while the marginal utility of pollution is harmful and decreasing, then

6 $$U = U(C(E) - P)\quad (U_{C} > 0,U_{P} < 0,U_{CC} < 0,U_{PP} < 0,C^{\prime} > 0,C^{\prime\prime} < 0).$$

The energy stock is S(t), and E(t) denotes the rate of energy use at any time point t. Assuming the pollution flow (P) is proportional to energy use (E) as α, and the EDCS (A) reduces emissions at a rate of β. In addition, the pollution decays with a rate δ, then

7 $$\dot{P} = \alpha E - \beta A - \delta P\quad (\alpha ,\beta > 0,0 < \delta < 1).$$

We assume that the implementation of the EDCS also consumes the energy stock (S). Consumption of energy by other economic activities will also reduce the energy stock. We set the coefficients all to − 1 for simplicity, then

8 $$\dot{S} = - A - E.$$

From the above assumptions, we can get

9 $$Max\int\limits_{0}^{T} {U(C(E),P)dt} ,$$

*s.t.*

10 $$\dot{P} = \alpha E - \beta A - \delta P,$$

11 $$\dot{S} = - A - E,$$

12 $$P(0) = P_{0} > 0,\;P(T) = 0\;\left( {\text{T is given}} \right),$$

13 $$S\left(0\right)={S}_{0}>0, S(T)\ge 0,$$

14 $$E \ge 0,\;0 \le A \le \hat{A}.$$

By constructing the Hamiltonian function

15 $$H = U(C(E),P) + \lambda_{P} (\alpha E - \beta A - \delta P) - \lambda_{S} (A + E).$$

The first-order optimization condition is that

16 $$\frac{\partial H}{{\partial E}} = U_{C} C^{\prime}(E) + \alpha \lambda_{P} - \lambda_{S} = 0,$$

17 $$\frac{\partial H}{{\partial A}} = - \beta \lambda_{P} - \lambda_{S} = 0.$$

From Eqs. (2) (3) (7) (8), it follows that

18 $${U}_{c}{C}_{\left(E\right)}{\prime}=-\frac{\alpha +\beta }{\delta }{U}_{P}.$$

Since ${U}_{c}>0,{U}_{P}<0,{C}{\prime}>0,\alpha >0,\beta >\text{0,0}<\delta <1$, the above equation holds.

The above equation shows that the EDCS can be most efficient when the utility generated by energy consumption is equal to the negative utility of pollution under the proportional trade-off between the EDCS and pollution attenuation.

Therefore, we put forward the following research hypothesis:

#### H1

The EDCS will cause a difference between the EEL of the pilot area and the non-pilot area, and continuously improve the EEL of the pilot area.

## Research design

### Sample selection and data sources

This article uses 2547-panel data from 284 prefecture-level cities from 2009 to 2017, in which the relevant data from Hong Kong, China, Macao, China, Taiwan, and Tibet are excluded. The data are mainly obtained from the Statistical Yearbook of China’s Cities, the Statistical Yearbook of China, the statistical yearbooks of each province, and the Statistical Bulletin on the National Economic and Social Development of Each Province and Municipality, which are analyzed by the hand-arranged by the authors.

Given that the EDCS in 2015 stipulates that the pilot provinces are Jilin, Jiangsu, Shandong, Hunan, Chongqing, Guizhou, and Yunnan, we treat the 63 cities in the pilot provinces as the experimental group, which is represented by a dummy variable with an assignment of 1, and the other cities in the non-pilot provinces are assigned a value of 0. The pilot period was started in 2015 and began to be promoted nationwide in 2018, so we set 2015–2017 as the implementation year of the EDCS, assigning a value of 1 to it, and 2009–2014 as the year before the release of the system, assigning a value of 0 to it. Finally, in order to prevent extreme outliers from interfering, all the continuous variables are subjected to the 1% Winsor.

### Variable selection and data description

The primary explanatory variable in this article is the EEL (Score). Moreover, the robustness analysis replaces the EEL with PM2.5.

Control variables, combined with Kaya^41^ constant equation, Grossman and Krueger^42^ environmental Kuznets curve, Ehrlich and Holdren^43^ IPAT model, the control variables selected in this study include GDP and the proportion of the secondary industry in GDP. In addition to the above variables, unlike the previous literature, this article also considers that industrial electricity consumption contributes to pollution emissions, which hurts the EEL, while the area of green space in urban parks can absorb harmful gases, which has a positive effect on the EEL. Therefore, this article includes the two variables of industrial electricity consumption and the area of green space in urban parks as the control variables. Therefore, this study includes two variables, namely, industrial electricity consumption and green space area of urban parks, as control variables.

The descriptive statistics of the main variables in Table 1 show that the mean value of ecological environment water is 11.1401, the standard deviation is 12.6839, the minimum value is 3.5058, the median is 7.7617, and the maximum value is 93.6743, which indicates that the performance of the EEL varies significantly among different samples.

**Table 1:** Descriptive statistics.

Variables	Mean	Std. dev	Min	Med	Max
Score	11.1401	12.6839	3.5058	7.7617	93.6743
PM2.5	44.3702	19.3508	10.3901	41.0109	93.7851
GDP	16.3066	0.9514	14.2603	16.2330	18.9238
Structure	48.7456	10.2085	20.5400	49.2300	73.5500
Elect	12.6703	1.3201	9.0325	12.7706	15.4307
Park	16.0922	2.0079	10.3578	15.8777	21.4380

The mean value of PM2.5 is 44.3702, the standard deviation is 19.3508, the minimum value is 10.3901, the median is 41.0109, and the maximum value is 93.7851, and the other explanatory variables have similar characteristics, which provide an objective basis and entry point for the relevant research on the EEL of the EDCS at a later stage.

### Model specification

This study applies the DID model to assess the EDCS’s effect on sewage and its degree of influence on the EEL. Relative to traditional panel data analysis, the DID method can avoid the endogeneity problem when policy is used as an explanatory variable. The DID model can control not only the unobservable individual heterogeneity among the samples but also the influence of unobservable aggregate factors that change over time, thus obtaining an unbiased estimation of the policy effect.

The EDCS is identified by comparing the EEL difference before and after the policy in the pilot areas and the non-pilot areas. Based on this, we set the following benchmark regression model:

$$Score_{it} = \beta_{0} + \beta_{1} * Treat_{it} * Post_{it} + \beta_{2} * X_{it} + \gamma_{i} + \lambda_{t} + province_{j} * year_{t} + \varepsilon_{it}$$

where *i* represents cities, and *t* represents years. *Score*_*it*_ is the explanatory variable for the EEL. *Treat*_*it*_ is a dummy variable used to identify the pilot cities, which equals 1 if a city is in the treatment group and 0 otherwise. *Post*_*it*_ is the time dummy variable before and after the implementation of the EDCS, which equals 0 for 2009–2014, and 1 for 215–2017. *Treat*_it_*Post_*it*_ is the core explanatory variable that this study focuses on examining, and *β*_*1*_ is the estimated causality coefficient that we try to identify. *X*_*it*_ represents a series of control variables, including GDP, the proportion of the secondary industry in the GDP (Structure), industrial electricity consumption (Elect), and the area of green space in urban parks (Park). *γ*_*i*_, *λ*_*is,*_ and provincej*yeart are the individual, time, and two-digit ISIC province fixed effects, respectively. *ε*_*it*_ is the random error term.

## Empirical results and robustness tests

### Test on policy effectiveness

#### Benchmark findings

Table 2 Main results on the effects of the EDCS reports the results of the benchmark model regression controlling for individual effect, time-fixed effect, and province effect (Province × Year) and introducing control variables such as GDP, the share of the secondary industry in GDP, industrial electricity consumption, and the green space area of urban parks. The explanatory variables in columns (1)–(4) are the logarithm of the EEL. Since the EEL is smaller after taking the logarithm, this study first expands the unit of the EEL by 100 times and then takes the logarithm when conducting the specific analysis. Columns (1)-(2) reflect the average treatment effect, and columns (3)-(4) reflect the dynamic impacts. From columns (1) and (2), it is found that under the influence of the EDCS, compared to the non-pilot provincial cities, the results from the average treatment effect show that the EEL in the pilot area, at the level of 1%, significantly increased.

**Table 2:** Main results on the effects of the EDCS.

Variables	Static effect		Dynamic effect	
	(1)	(2)	(3)	(4)
Treat × post	0.441***	0.483***		
	(0.0000)	(0.1544)		
Treat × year2016			88.801***	72.562***
			(0.0000)	(4.9234)
Treat × year2017			0.035	0.121
			(.)	(0.0895)
GDP		1.904***		1.904***
		(0.6384)		(0.6384)
Structure		− 0.058*		− 0.058*
		(0.0316)		(0.0316)
Elect		− 0.419**		− 0.419**
		(0.1832)		(0.1832)
Park		1.465***		1.465***
		(0.4434)		(0.4434)
Constant	11.132***	− 34.174***	11.222***	1.904***
	(0.2197)	(12.0919)	(0.2197)	(0.6384)
Year FE	Yes	Yes	Yes	Yes
Individual FE	Yes	Yes	Yes	Yes
Province × year	Yes	Yes	Yes	Yes
Observations	2547	2547	2547	2547
R-squared	0.466	0.545	0.466	0.545

Data in parentheses in the table are standard errors.

*p < 0.1, **p < 0.05, ***p < 0.01; Robust standard errors are clustered at the city-level.

In the dynamic effect, the EEL of the unit pilot area shows a meaningful influence during the implementation of the EDCS. The EEL of the unit pilot area significantly increased in 2016.

The regression results of the control variables show that the EEL sharply declines with the increase of the proportion of the secondary industry in GDP, showing that industrial pollution is the key concern of the EEL. Additionally, industrial thermal power consumption also hurts the ecological environment due to the production of industrial waste gas such as SO_2_. There is a highly positive correlation between the increase in GDP and the EEL because the regions with fast economic development in China pay more attention to the EEL, and the GDP also improves the EEL to a certain extent. The green space area of urban parks plays a positive role at the EEL, absorbing harmful gases, releasing more oxygen, and having the green island effect. Therefore, the EDCS has a dramatic benefit on the EEL of the areas in the sample.

#### Parallel trend assumption

**Fig. 2:**
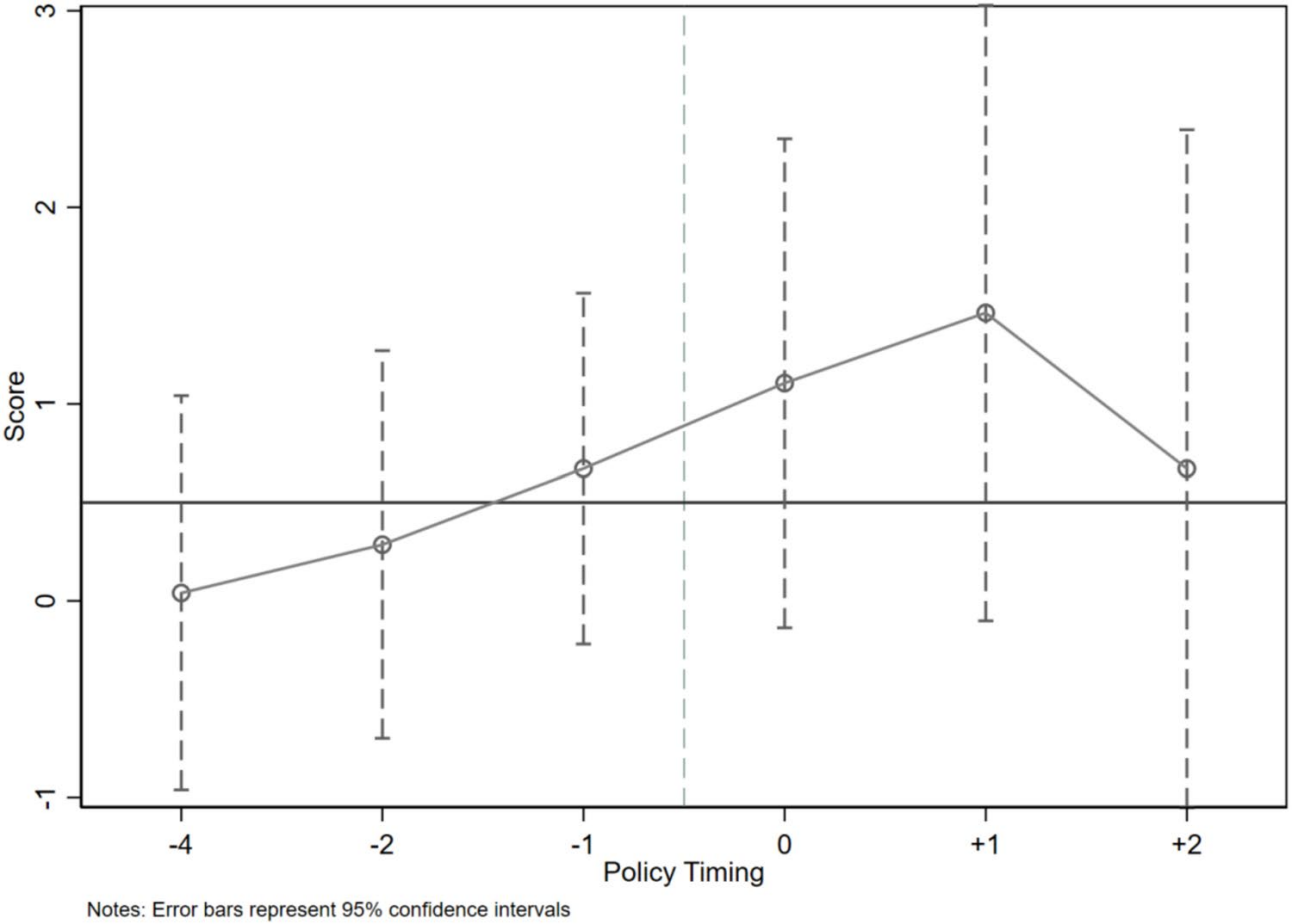
The parallel trend test of the EDCS.

The validity of the estimation results of the DID model relies on whether the parallel trend assumption is satisfied, i.e., whether the pilot area and the control group area have similar characteristics and trends before implementing the EDCS. This study draws on the treatment of Shi and Li^44^, using 2015, the pilot year of the EDCS, as the base year. Then, a separate OLS-DID regression consistent with the primary regression is conducted on the explanatory variables for the first three years and the last 2 years of the base year. Eventually, there is no significant difference between the EEL of the treatment group and the control group before the EDCS in Fig. 2, and the above results indicate that the DID in this article satisfies the parallel trend assumption.

The dynamic effect of the parallel trend test shows that the EEL shows a rapid increase in the first year after the base year, and there is no continuous effect.

### Endogeneity test

Due to data limitations, omitted variables may exist, potentially introducing endogeneity into the regression analysis. To address this issue, we employ a partially linear instrumental variable model based on Double Machine Learning (DML), following Chernozhukov et al.^45^. The DML combines regularization techniques with multiple machine learning algorithms, enabling the automatic selection of effective control variable combinations. This approach offers significant advantages in variable processing and model estimation.

Using this framework, we split the sample in a 1:2 ratio and conducted regression tests. The results in Columns (1) and (2) of Table 3 reaffirm our baseline conclusions.

**Table 3:** Results of endogeneity test.

Variables	(1)	(2)
DID	0.790***	0.740***
	(0.244)	(0.252)
Linear control variables	Yes	Yes
Quadratic control variables	Yes	Yes
Time fixed	Yes	Yes
City fixed	Yes	Yes
N	2547	2547

### Robust analysis 

#### Alternative indicator

In this article, the PM2.5 is used as a proxy variable for the dependent variable to replace the EEL, which further verifies the effect of the EDCS. Where the PM2.5 data comes from the Atmospheric Composition Analysis Group of Dalhousie University, the concentration means data after matching the vector maps of prefecture-level cities through raster processing. The column (2) test results in Table 4 show that the EDCS reduces PM2.5 at the 1% significance level, indicating that the findings of this article will be strongly robust.

**Table 4:** Robustness analyses: Alternative indicator.

Variables	(1)	(2)
Treat × post	− 1.318	− 1.458***
	(5.8147)	(0.1341)
Constant	55.770***	46.784***
	(3.8455)	(8.4656)
Controls	No	Yes
Year FE	Yes	Yes
Individual FE	Yes	Yes
Province × year	Yes	Yes
Observations	2547	2547
R-squared	0.862	0.862

Data in parentheses in the table are standard errors.

*p < 0.1, **p < 0.05, ***p < 0.01; Robust standard errors are clustered at the city-level.

#### Placebo test

**Fig. 3:**
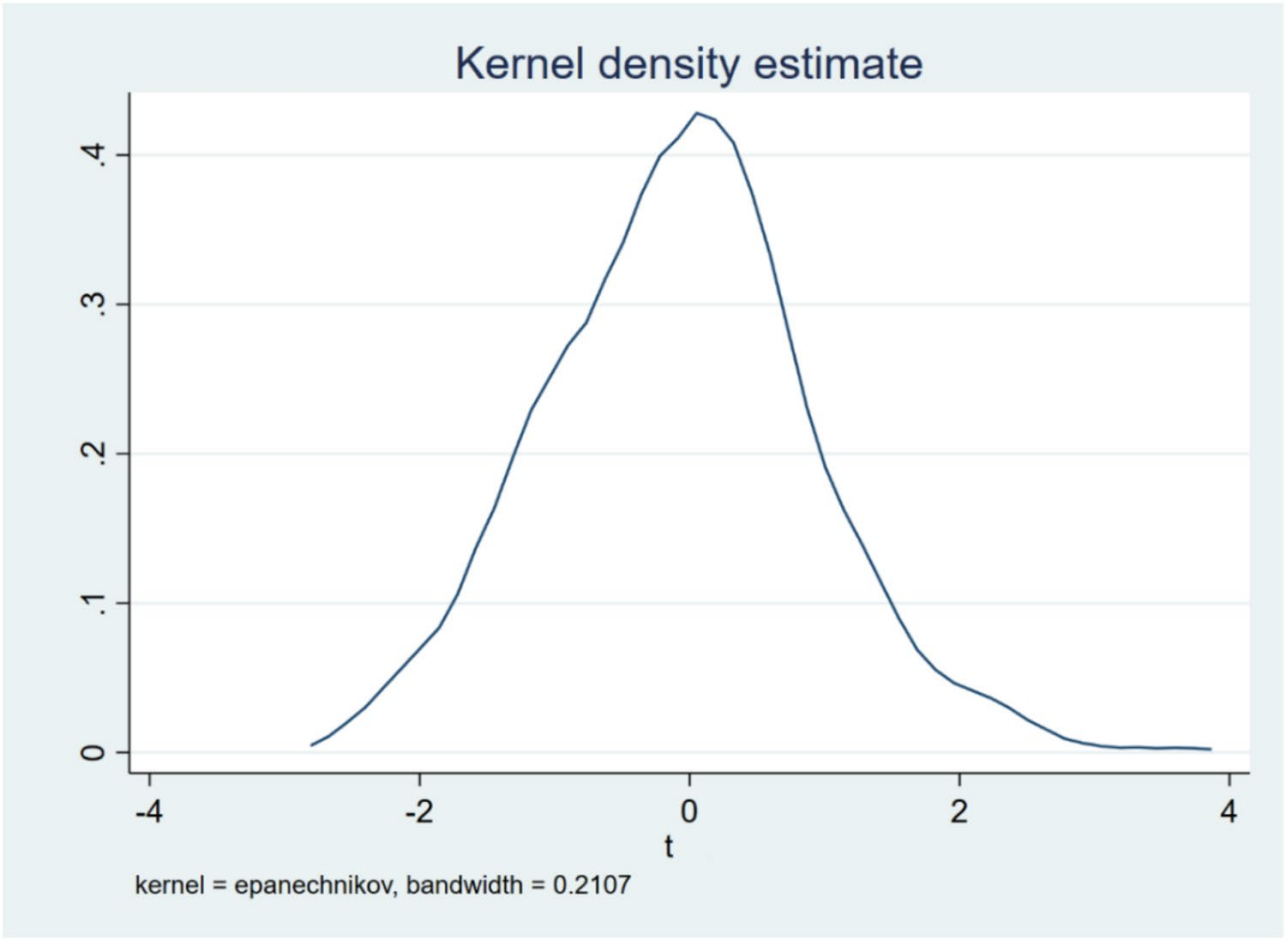
The Placebo test of the EDCS.

In order to further exclude the influence of other unobservable factors and ensure that the EDCS is a causal factor at the EEL, this study conducts a placebo test by randomly assigning pilot provinces. Specifically, 1000 random samples are taken from 284 cities in all 30 provinces in the sample, from which 63 cities are randomly selected as the treatment group and assumed to have implemented the EDCS. The remaining 121 cities are used as the control group in each sample. According to the baseline model regression again, the kernel density distribution of the explained variable of the EEL (see Fig. 3) shows that most of the sampling of the estimated coefficients of t-value less than 2, P-value is greater than 10%, which means that the results of the random sampling are not significant. Therefore, it can be concluded that the result passes the placebo test and that the impact of the EDCS on the EEL in the pilot area is not driven by other unobservable factors.

#### Propensity score matching (PSM) estimation

Due to the use of national city samples, there are many sample differences between cities, and in order to avoid the systematic differences and the risk of estimation bias^46^ in DID and to try to make the experimental group and control group as similar as possible in terms of their characteristics before the policy is implemented, this study further adopts the propensity score matching difference-in-difference (PSM-DID) method to conduct a robustness test.

Table 5 shows the regression results of the PSM sample, and the EDCS can effectively improve the EEL, which further argues for the robustness of this study’s findings.

**Table 5:** PSM-DID results of the EDCS.

Variables	(1)	(2)
Treat × post	12.790***	11.156***
	(0.5427)	(0.7685)
Constant	9.353***	− 38.081***
	(0.2428)	(12.8810)
Controls	No	Yes
Year FE	Yes	Yes
Individual FE	Yes	Yes
Province × year	Yes	Yes
Observations	2295	2295
R-squared	0.464	0.540

Data in parentheses in the table are standard errors.

*p < 0.1, **p < 0.05, ***p < 0.01; Robust standard errors are clustered at the city-level.

#### Difference-in-difference-in-differences (DDD)

In order to exclude the interference of other policies so as to avoid the bias of estimation results caused by other policies in pilot and non-pilot areas, this study adopts the DDD method to overcome this problem. For example, in 2013, the State Council issued the ‘National Sustainable Development Plan for Resource-based Cities (2013–2020)’, which specifies 126 resource-based cities. Therefore, it can be grouped by resource-based cities, provincial capital cities, and key cities in the province, respectively, the DID results of each sub-sample, and then the differential. Ultimately, it can effectively eliminate the interference of other policies and other favorable factors in the study and get the net effect of the EDCS. Specific approach: Set up a new variable DDD; if the pilot cities belongs to the resource cities, the provincial capital cities, or the pilot of the key cities of the province, then assign a value of 1 to it, otherwise 0.

The final experimental results (see Table 6) show that the EDCS effectively improves the EEL, and the estimated coefficients are all at a 1% significance level. Ultimately, it effectively supports our previous research conclusions.

**Table 6:** The results of the DDD method.

Variables	(1)	(2)
DDD	3.565***	3.178***
	(1.0409)	(1.0128)
Constant	10.650***	− 36.005***
	(0.2424)	(11.8141)
Controls	No	Yes
Year FE	Yes	Yes
Individual FE	Yes	Yes
Province × year	Yes	Yes
Observations	2547	2547
R-squared	0.476	0.553

Data in parentheses in the table are standard errors.

*p < 0.1, **p < 0.05, ***p < 0.01; Robust standard errors are clustered at the city-level.

#### Robustness tests with adjusted policy timing

When selecting the timing for policy implementation in DID analyses, it is standard practice to base the timing on the official announcement of pilot programs by national authorities, particularly when policies are rolled out in batches at the national level. Studies typically use the year of the national announcement as the starting point for policy impact, rather than the specific implementation dates at the local level. For example, Ren et al.^16^ used 2007 as the baseline year for analyzing SO_2_ emission trading pilots, and Peng and Zhao^47^ directly adopted the national pilot announcement year for their study on rural collective property rights reform, without accounting for local implementation differences.

Given that the EDCS in pilot regions was implemented two years after the national announcement, we account for the delay between policy announcement and actual enforcement in our robustness checks. As shown in Table 7, the results remain consistent with our baseline conclusions.

**Table 7:** Robustness tests with adjusted policy timing.

Variables	(1)	(2)
DID	0.008*	0.756*
	(0.0041)	(0.4063)
Controls	Yes	Yes
Time fixed	Yes	Yes
ID fixed	Yes	Yes
N	2543.000	2543.000
Adj-R^2^	0.931	0.931

Data in parentheses in the table are standard errors.

*p < 0.1, **p < 0.05, ***p < 0.01; Robust standard errors are clustered at the city-level. The score in column (1) is not multiplied by 100.

#### Additional control variables

To account for potential factors influencing urban ecological quality, we include merchandise exports as a proxy for international trade and local fiscal expenditures within the general budget as a measure of local fiscal support. Even after controlling for these variables, the regression results remain robust in Table 8.

**Table 8:** Results with additional control variables.

Variables	(1)	(2)
DID	0.469***	0.472***
	(0.1557)	(0.1613)
Export	− 0.320	− 0.321
	(0.2656)	(0.2666)
Fiscalexp		0.114
		(1.1227)
Other control	Yes	Yes
N	2538.000	2538.000
Adj-R^2^	0.547	0.547

Standard errors in parentheses, *p < 0.1, **p < 0.05, ***p < 0.01.

### Heterogeneity analysis

The current resource development and ecological environmental protection are generally unbalanced and uncoordinated. In some areas, the intensity of resource development is high, the level of comprehensive utilization is low, and three high (high energy consumption, high pollution, high emission) projects abound, causing severe damage to the ecological environment. Resource-based cities have impacts on environmental protection policies due to differences in the level of resource endowment^48^, so it is of great practical significance to study the heterogeneous policy effect of the EDCS on resource-based cities and non-resource-based cities. Based on the Circular on the National Sustainable Development Plan for Resource-based Cities (2013–2020), which defines resource-based cities in China^49^, this article divides the sample area into two categories of resource-based and non-resource-based cities and conducts regressions by sub-sampling. The results of the analysis are reported in Table 9, where columns (1) and (2) show the policy effect of the EDCS on the EEL in resource-based cities, and columns (3) and (4) reflect the policy effect in non-resource-based cities.

**Table 9:** Heterogeneity analyses.

Variables	Resource-based cities		Non-resource-based cities	
	(1)	(2)	(3)	(4)
Treat × post	0.441***	0.577***	0.769	− 2.445
	(0.0000)	(0.1094)	(0.8158)	(1.8941)
Constant	9.424***	20.467***	10.802***	− 48.932***
	(0.1340)	(7.3447)	(2.3674)	(16.6626)
Controls	No	Yes	No	Yes
Year FE	Yes	Yes	Yes	Yes
Individual FE	Yes	Yes	Yes	Yes
Province × year	Yes	Yes	Yes	Yes
Observations	1081	1081	1467	1467
R-squared	0.911	0.916	0.221	0.385

Data in parentheses in the table are standard errors.

*p < 0.1, **p < 0.05, ***p < 0.01; Robust standard errors are clustered at the city-level.

It is easy to find that the EDCS significantly improves the EEL in resource cities. The effect on the EEL in non-resource cities is not significant, which may be due to the fact that non-resource cities have little environmental protection pressure, and the EDCS does not have a substantial impact on them.

### Mechanism analysis

Previous analyses indicate that, compared to the control group, the EDCS significantly improved ecological quality in treatment regions. A natural follow-up question is: *Through what channels does the EDCS influence ecological quality?* This section explores this question by examining two potential pathways: Industrial structure transformation and Technological innovation.

### Industrial structure

The EDCS is a form of environmental regulation that can effectively compel regions to undergo industrial transformation^48^. Industrial upgrading can break the “resource curse” and promote positive environmental outcomes^50^. As a key indicator of green development, ecological quality is influenced by industrial transformation, as demonstrated by Han et al.^51^, who measured the impact of industrial transformation on green development using total factor productivity. Thus, the EDCS may influence ecological quality through industrial transformation.

To test this pathway, we follow Gan et al.^52^ and use the proportion of secondary and tertiary industry output, as defined by Clark’s Law, to measure industrial structure upgrading. Our empirical design incorporates this metric to conduct mechanism tests.

After controlling for these variables, the baseline regression results in Table 10, Columns (1) and (2), provide insights into the moderating effects of industrial structure. Column (1) includes an interaction term between the EDCS and industrial structure, revealing a significant positive effect on regional ecological quality. This suggests that the EDCS’s impact is a combined effect of the policy and industrial structure. Column (2) introduces an interaction term between the EDCS and industrial upgrading, which also shows a significant positive effect, indicating that the policy’s influence is further amplified through industrial upgrading.

**Table 10:** The results of the moderation tests.

Variables	(1)	(2)	(3)
DID*Structure1	33.046***		
	(11.5433)		
DID*Structure2		55.042***	
		(13.6054)	
DID*Invent			0.001***
			(0.0003)
DID	− 11.714***	− 65.918***	-0.378
	(4.0088)	(16.1288)	(0.4782)
Structure1	− 11.631*		
	(6.9941)		
Structure2		− 20.154*	
		(11.4675)	
Invent			0.002***
			(0.0006)
N	2531.000	2531.000	2531.000
Adj-R^2^	0.137	0.140	0.158

Data in parentheses in the table are standard errors.

*p < 0.1, **p < 0.05, ***p < 0.01; Robust standard errors are clustered at the city-level.

**Fig. 4:**
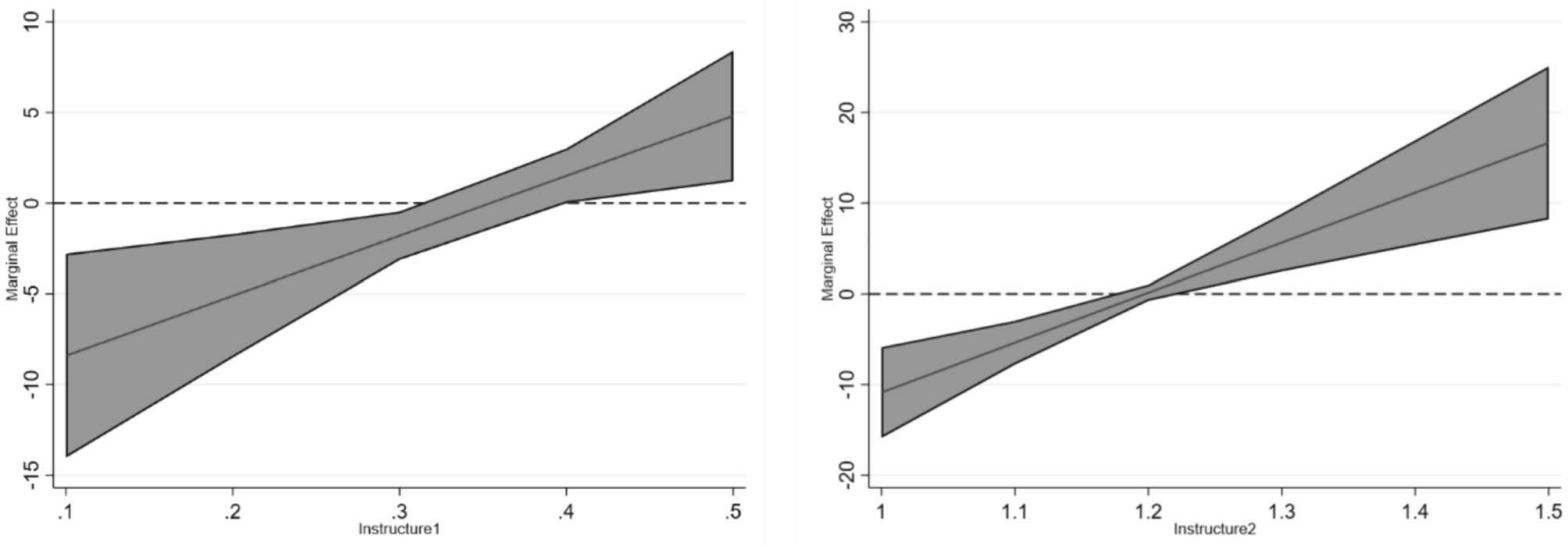
Average marginal effect plots (structure1 and structure2).

Figure 4 shows the nonlinear characteristics of the marginal contribution of industrial structure. The left figure shows that when the industry level is low (lnstructure1-w < 0.3), the policy effect is not significant; When the industrial upgrading reaches the threshold (lnstructure3w_ ≈ 0.4), the policy effect significantly increases, showing an accelerated growth pattern. The right figure shows that when the level of industrial upgrading is low (lnstructure2 < 1.2), the policy effect is not significant; When the industrial upgrading reaches the threshold (lnstructure2 ≈ 1.3), the policy effect significantly increases, showing an accelerated growth pattern.

### Technological innovation

The EDCS can stimulate an “innovation compensation” effect^11^, promoting regional technological progress and thereby supporting the Porter Hypothesis. As pollution control technologies advance, they can positively influence ecological quality. Cai et al.^53^ used the number of patent authorizations as a measure of corporate innovation. Drawing on this approach, this study examines whether the EDCS enhances regional EEL through the lens of authorized invention patents. Specifically, we use the total number of authorized invention patents, log-transformed for analysis.

Table 10 Column (3) shows that, after controlling for other variables, the interaction term between the EDCS and technological innovation has a significant positive effect on regional EEL. This indicates that the EDCS positively influences the EEL through innovation-driven pathways.

**Fig. 5:**
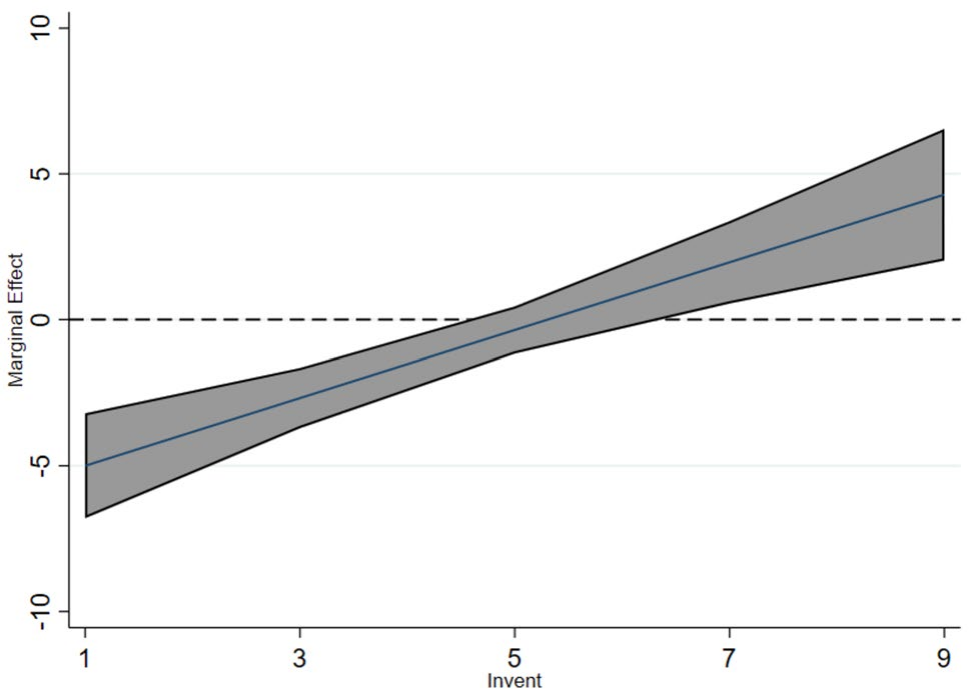
Average marginal effect plots (invent).

Figure 5 reveals the moderating effect of technological innovation. When at a lower level of innovation (Invent = 3), the policy effect is not significant, but when innovation is at a high level, the policy effect significantly increases, showing an accelerated growth pattern.

## Conclusions and implications

In the context of the pilot EDCS in 2015, this article empirically investigates the impact of the EDCS on the emission reduction effect and the EEL based on 2547-panel data of 284 cities from 2009 to 2017. After empirical analysis, this study draws the following conclusions: (1) Under the impact of the EDCS, the pilot area has an obvious EEL after implementing the pilot policy. (2) The EDCS has a dynamic effect, resulting in a rapid increase in the EEL 1 year after the base year. (3) Furthermore, the EEL is also heterogeneous: the effect of resource cities is significant, while the effect of involuntary cities is not significant. (4) Industrial structure transformation and technological innovation serve as crucial mediating variables, enabling the EDCS to significantly influence the EEL. Based on the above findings, we provide the following recommendations.

Firstly, the sustained emission reduction effect cannot be separated from the policy effect but also requires the active participation of market players so as to achieve the combination of top-down and bottom-up, and the joint role of efficient market and active government, which will work together to explore the energy saving and consumption reduction path.

Secondly, the impact on different types of resource cities, to form a dynamic adjustment mechanism and ultimately reach the resource cities and non-resource cities under the influence of the policy, is to realize the purpose of emissions reduction and enhance the EEL.

Finally, it is necessary to have a professional division of labor, multi-party cooperation, integrated policies and personnel training, active assessment and timely monitoring, and other means to provide a strong guarantee for improving the EEL.

## Supplementary Information

Supplementary Information.

## Data Availability

Data is provided within the manuscript or supplementary information files.
