# Supplementary material for: Assessing the impact of the eco-environmental damage compensation system
Source: Sci Rep. 2025 Aug 19;15:30407. doi: 10.1038/s41598-025-12241-x (PMC12365327; doi:10.1038/s41598-025-12241-x)
Supplement: Supplementary file 1 — Supplementary Information. [file 41598_2025_12241_MOESM1_ESM.docx]

**Appendix**

We use the entropy weight method to determine the weights of each indicator, and the final weight allocation is detailed in Table A.1. The results show that air pollution (with a primary weight of 40.79%) has the greatest impact on EEL, with emission systems and air pollution being key negative limiting factors. For each secondary indicator (i.e. the object directly assigned weight by entropy weight method), we independently increase its weight by 10% (+10%) and decrease it by 10% (-10%), and recalculate the EEL comprehensive evaluation value of all samples/units. The sensitivity analysis results indicate that within the disturbance range of ± 10% of the secondary indicator weight, the EEL comprehensive value calculated based on the entropy weight method shows good robustness.

**Table Appendix 1 Weight Distribution Table of Entropy Weight Method**

| **Level 1 Indicator** | **Level 1 Weight** | **Level 2 Indicator** | **Level 2 Weight** | **Combined Weight** | **Direction** |
| --- | --- | --- | --- | --- | --- |
| Greenarea System | 4.50% | Greenarea | 100.00% | 4.50% | (+) |
| Park Services | 15.24% | Park | 100.00% | 15.20% | (+) |
| Emission System | 39.47% | So2 | 61.25% | 24.18% | (-) |
|  |  | nox | 38.75% | 15.29% | (-) |
| Air Pollution | 40.79% | PM25 | 53.63% | 21.89% | (-) |
|  |  | Dust | 46.37% | 18.91% | (-) |

Notes: *Combination weight= Level 1 weight * Level 2 weight*; Sensitivity analysis based on Level 2 weight with ± 10% perturbation.
